# A novel method to identify pre-microRNA in various species knowledge base on various species

**DOI:** 10.1186/s13326-017-0143-z

**Published:** 2017-09-20

**Authors:** Tianyi Zhao, Ningyi Zhang, Ying Zhang, Jun Ren, Peigang Xu, Zhiyan Liu, Liang Cheng, Yang Hu

**Affiliations:** 1 Department of Computer Science and Technology, Harbin Institute of Technology, Harbin, 150001 People’s Republic of China; 2 Department of Pharmacy, Heilongjiang Province Land Reclamation Headquarters General Hospital, Harbin, 150088 China; 3 School of Life Science and Technology, Harbin Institute of Technology, Harbin, 150001 People’s Republic of China; 4 College of Bioinformatics Science and Technology, Harbin Medical University, Harbin, 150001 China

**Keywords:** Pre-miRNA identification, BP neural network, Adaboost

## Abstract

**Background:**

More than 1/3 of human genes are regulated by microRNAs. The identification of microRNA (miRNA) is the precondition of discovering the regulatory mechanism of miRNA and developing the cure for genetic diseases. The traditional identification method is biological experiment, but it has the defects of long period, high cost, and missing the miRNAs that but also many other algorithms only exist in a specific period or low expression level. Therefore, to overcome these defects, machine learning method is applied to identify miRNAs.

**Results:**

In this study, for identifying real and pseudo miRNAs and classifying different species, we extracted 98 dimensional features based on the primary and secondary structure, then we proposed the BP-Adaboost method to figure out the overfitting phenomenon of BP neural network by constructing multiple BP neural network classifiers and distributed weights to these classifiers. The novel method we proposed, from the 4 evaluation terms, have achieved greatly improvement on the effect of identifying true pre-RNA compared to other methods. And from the respect of identifying species of pre-RNA, the novel method achieved more accuracy than other algorithms.

**Conclusions:**

The BP-Adaboost method has achieved more than 98% accuracy in identifying real and pseudo miRNAs. It is much higher than not only BP but also many other algorithms. In the second experiment, restricted by the data, the algorithm could not get high accuracy in identifying 7 species, but also better than other algorithms.

## Background

MicroRNAs(miRNAs) are a class of small single-strand and non-coding RNA molecules of approximately 22 nucleotides in length, miRNAs play important roles in many biological process including affecting stability, metabolism, signal translation, disease development and translation of mRNAs [1]. Meanwhile, miRNAs are also very important in the treatment of diseases, such as: cancer [2], X chromosomal defects [3], DiGeorge disease [4], etc. As the development of science and technology, people pay more and more attention to miRNA research, amount of novel miRNAs are discovered, the number and functional features are far beyond our imagination [5, 6]. The main challenge of studying miRNAs is how to find miRNAs and the action sites, at present the main methods for identifying miRNAs are cDNA clone and sequencing and computational prediction, the expression of cDNA sequencing method is low and costs amount of time and funding [7–12]. Therefore, computational method are more prevalent, several algorithms have been proposed to detect pre-miRNAs, the main challenge is to discriminate the real pre-miRNAs from the pseudo ones and identify novel miRNAs.

Recently, for miRNAs identification, machine learning techniques have been widely used. Sequence composition and structural conformation features are applied to train the learning system, then the classifiers employ multiple features to obtain the final prediction. Xue et al. discovered the significant difference of local contiguous sub-sequence between real and pseudo miRNAs. Therefore, they applied the three-character group local structure-sequence features to describe the samples, and based on SVM they proposed the triplet-SVM to identify novel miRNAs and miRNAs from specific species [13]. Zhao et al. employed parallel triplet local structure-sequence feature, however they chose the first nucleotide of the contiguous triplet group as the local structure-sequence feature, and add two MFE related features and two nucleotide pairing features, then apply the SVM classifier PMirP [14]. Jiang et al. added MFE and *P*-value as the features based on the feature set in [13], and proposed the classifier Mipred based on RF method [15], Limin Jiang. et [16] applied BP neural network to identify real and pseudo pre-miRNAs, and proved the superiority of BP neural network by comparing with triplet-SVM、RF methods.

Neural network [17] and other classifiers of data driving tend to occur overfitting phenomenon. BP neural network is a widely used classification algorithm, it has strong self-learning ability and is particularly suitable for solving internal mechanism problems. However, the algorithm tends to be overfitting and the output is unstable. The boosting algorithm [18] integrates multiple weak classifiers to obtain a strong classifier and avoid overfitting phenomenon. Freund [19] promoted the boosting algorithm to Adaboost(adaptive boosting) so that the new algorithm can be more suitable for practical applications.

Therefore, in this study, we proposed BP-Adaboost algorithm to establish multiple BP neural network classifiers and distribute the weights of classifiers through Adaboost framework. Eventually a strong classifier with high accuracy is obtained.

## Methods

### Feature extraction

#### N-gram frequency

In the recent years, for pre-miRNA identifying, studies have shown that the local primary sequence is crucial to the pre-miRNA sequence [20]. Therefore, the n-gram frequency is the most commonly used feature in the primary sequence feature selecting [21, 22]. However, there is still no exact criteria for choosing the value of n. Thus, n is often chosen by comparing the effect of n-gram frequency with different n-values. In this study, we chose n as 3. Thus, for a certain sequence, there are 64(43) combinations in a triple-nucleotide group, then we computed the frequency occurrence of these 64 combinations in the sequence.

### Energy characteristics features

Some studies showed that the minimum free energy (MFE) indicates the stability of a secondary structure. The real pre-miRNA sequences have a lower minimum free energy than that of the randomly generated pre-miRNA sequences. Therefore, the minimum free energy of a pre-miRNA sequence is also considered as a feature in distinguishing the pre-miRNA sequences. RNAfold is used to compute the MFE value of a secondary structure.

### Structural-diversity based features

The base-pair of nucleotide in the sequences is also a remarkable characteristic in distinguishing real and pseudo pre-miRNAs. The traditional nucleotide pairing are A-U pairing and C-G pairing, but in pre-miRNA sequences there are also other forms of nucleotide pairing, such as the G-U pairing. Therefore, in this study, the G-U pairing is also included as one of the features.

### Triple structure sequence

To highly specify the primary sequence features, the secondary structure is also a significant feature. The software RNAfold is employed to calculate the potential secondary structure. In the predicted secondary structure, there are two states for each nucleotide of the sequence, matched or non-matched, indicated by brackets ‘(’ or ‘)’, and dots ‘.’. In this study, the two brackets are not distinguished, which means every ‘)’ is replaced by ‘(‘. For any three nucleotides groups, there are 8 (2×3) possible characters combinations, including ‘(((’, ‘((.’, ‘(.(’, ‘(..’, ‘.((’, ‘.(.’, ‘..(’ and ‘…’. Considering the first nucleotide of the three characters group, then there are 32 (4 × 8) different combinations, which are denoted as ‘A(((’, ‘U(..’, etc. For a given sequence, the 32 dimensional feature vector is sufficient information for miRNA identification. Then the calculated 32D feature is employed to train the classifier.

Using the feature extraction methods which we mentioned above, we can extract 98D features from any pre-miRNA sequence in total.

First we integrate the obtained pre-sequence into primary sequence and secondary structure sequence. For the primary sequence, we choose the n-gram parameter(*n* = 3) and extracted 64 dimensional feature. In addition to calculating the potential structure, the software RNAfold is applied to predict the second structure sequence. In this structure, we extracted 32 dimensional features according to the triple structure sequence. We also extract the energy characterization MFE as a feature of pre-miRNA sequence. The possible nucleotide pairing G-U is included as the last feature. Therefore, altogether we extracted 98 features.

**Fig. 1:**
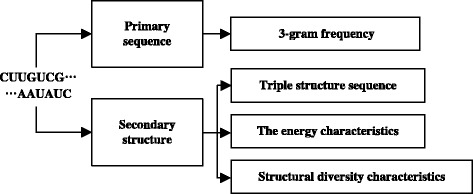
Flow chart of Feature extraction

The Flow chart of Feature extraction is illustrated in Fig. 1.

### Methods and framework

#### BP-Adaboost

Due to BP neural network tends to be caught in overfitting phenomenon and unstable output, in this study, we proposed a new method BP-Adaboost based on BP neural network. We employed BP neural network as a weak classifier to establish multiple classification model by training repeatedly. Finally, a strong classifier is obtained after adjusting the weights through Adaboost.

**Fig. 2:**
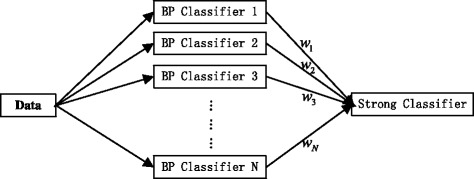
Frame of BP-Adaboost

The framework is shown in Fig. 2.

First, we establish N BP network classifiers by the extracted features and their corresponding labels. While training and establishing classifiers, each classifier will get a corresponding weight. In the end, we obtained a strong classifier by combining these N weight-distributed classifiers.

### The construction of BP neural network classifier

To accomplish the construction of classifier, we also need to set the various parameters besides the obtained features and the corresponding labels.

First, we need to choose the number of nodes in the hidden layer, since there is no specific criterion at present, we choose the number through the empirical formula as followed.

1 $$ M=\sqrt{N+L}+a $$

The number of nodes(M) equals to a constant(*a* ∈ [1, 10]) plus the square root of the number of feature dimensions(N) plus the output(L). In this study, *N* = 98, L = 1. From the formula above, in this study, we choose M = 12.

After setting the number of nodes (12) and hidden layers(3) of the BP neural network, the structure of BP network is 98–12-1. Then set the paramaters (Epochs, The learning rate, Error bounds) and functions(Performance function, Transfer function of hidden layer nodes, Transfer function of output nodes,The training function) of BP network. The parameters and functions of BP network are listed in Table 1.

**Table 1:** Parameters and functions of BP neural network

Setting items	The value set
Epochs	50
The learning rate	0.1
Performance function	MSE
Error bounds	0.01
Transfer function of hidden layer nodes	Tansig
Transfer function of output nodes	Purelin
The training function	Trainlm

### Method process

For a given set of multiple classification training data *T* = {(*x*_1_, *y*_1_),  ⋯ , (*x*_*N*_, *y*_*N*_)}, the input data *x*_*N*_ ∈ *X* ⊆ R^n^, with an arbitrary integer label *y*_*N*_. First, initialize the weight distribution, the initial weight of each sample is 1/N. Then train the sample to get the first classifier, and reduce the weight of the correct classification samples while raising the weight of improper classification samples. By the statistics of the weights of improper classification samples, the weights of corresponding classifiers are obtained. Repeat the process above, we can get multiple classifiers and the corresponding weights, then the ultimate strong classifier are obtained. The method process is as followed,
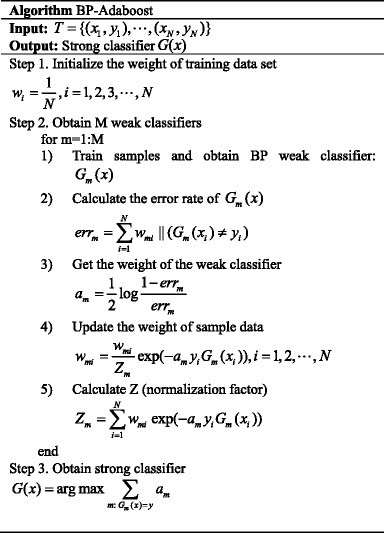


## Results

### Data description

The dataset of pre-miRNAs was downloaded from http://bioinf.sce.carleton.ca/SMIRP [23]. There are 7 species samples in the data set and each species has both a positive sample set and a negative sample set.

These 7 species are *Anolis carolinensis*、*Arabidopsis lyrata*、*Arabidopsis thaliana*、*Drosophila melanogaster*、*Drosophila pseudoobscura*、Epstein barrvirus、Xenopus tropicalis. As each species has a negative sample set, altogether we obtain 8 classes of pre-miRNAs, one of them is pseudo pre-miRNAs. The total number of the gene sequences of the whole data set is 12,846, among them 9264 sequences are pseudo pre-miRNAs, and the rest 3582 of them are true pre-miRNAs.

In this article, we distinguish the real pre-miRNAs from the pseudo ones before classifying these 7 species. *V*-fold cross-validation with moderate computational complexity is widely used for model selection. Usually, a value of *V* between 5 and 10 is selected based on experience. In this study, V = 10. First, the 12,846 sequences are randomly divided into 10 groups, and choose 9 of them to be training samples. The last one is tested as the testing set for a total of 10 training times. The final statistical results are averaged.

### Evaluation criteria

The four kinds of prediction results are true positive (TP), false positive (FP), true negative (TN), and false negative (FN). Many evaluation indicators can be used for the classification results. First, the accuracy rate (ACC) is the proportion of the correct classification. Precision and recall are common used evaluation criteria in pattern recognition, precision represents the proportion of true positive samples of the classified positive samples, and recall represents the proportion of correctly classified positive samples of the whole positive samples, specificity represents the proportion of the correctly classified negative samples of the whole negative samples, the computational formula is as follows,

2 $$ ACC=\frac{TP+ TN}{TP+ FP+ TN+ FN} $$

3 $$ precision=\frac{TP}{TP+ FP} $$

4 $$ recall=\frac{TP}{TP+ FN} $$

5 $$ specificity=\frac{TN}{TN+ FP} $$

### The authentic classification of pre-miRNAs

In this study, the label of pseudo pre-miRNAs is 0, and the label of real pre-miRNAs is 1(for all species).

**Fig. 3:**
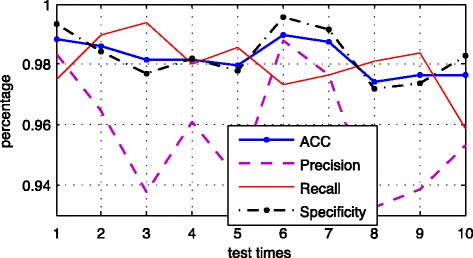
Results of 4 error standards of ten experiments

Figure 3 shows the curves of the four error metrics for 10 experiments, blue dot-solid line is the ACC curve, purple dotted line is precision curve, red solid line is recall curve, and black dash-dotted line is specificity curve. It is observed that the fluctuation of the precision curve is relatively large, and the rest three curves are more stable.

The error statistics of the average results of 10 experiments are shown in the Table 2. The table shows that BP-Adaboost algorithm is superior to other 4 algorithms in these 4 accuracy assessment, and Naïve Bayes is the worst. The accuracy of BP-Adaboost algorithm reaches 98.22%, and this represents the superiority and effectiveness of this novel method we proposed in distinguishing real and pseudo mi-RNAs. The table also shows the accuracy of BP neural network is only second to BP-Adaboost algorithm, and that’s the reason why we choose the BP neural network combined with Adaboost algorithm. Due to the randomness of BP network, by weighting multiple classifiers to obtain the final results effectively improves the classification accuracy and stability.

**Table 2:** Comparison of the BP-Adaboost with alternative models

Algorithm	ACC	Precision	Recall	Specificity
BP-Adaboost	0.9822	0.9576	0.9797	0.9830
BP	0.9541	0.9429	0.9736	0.9800
Random Forest	0.9336	0.9270	0.9744	0.9772
Naïve Bayes	0.7026	0.4831	0.9721	0.5987
SVM	0.8811	1	0.5729	1

### Species classification of pre-miRNAs

**Fig. 4:**
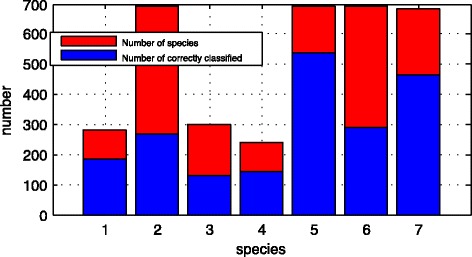
Results of 7 species classify by BP-Adaboost

The Fig. 4 shows the classification results from the statistic of each classified real pre-miRNA sequence.

There are 7 species in the graph, red bar shows the true number of the species, blue bar shows the correctly classified number. From the graph we can tell the accuracy of the classification of some species are not ideal, the reason is the number of pseudo pre-miRNA sequences is large. In the cross validation, real pre-miRNA sequences of the samples are not enough, so the number of real pre-miRNA sequences is smaller after the classification. Therefore, the samples of some kind of species are more likely not enough.

The classification accuracy of each species is shown as Table 3.

**Table 3:** Accuracy’s comparison of the BP-Adaboost with alternative models in 7 species

Species	BP-Adaboost	BP	RF	Naïve Bayes	SVM
*Anolis carolinensis*	0.66	0.10	0.78	0.69	0.14
*Arabidopsis lyrata*	0.39	0.25	0.53	0.21	0
*Arabidopsis thaliana*	0.45	0.23	0.67	0.54	0
*Drosophila melanogaster*	0.61	0.20	0.51	0.75	0.21
*Drosophila pseudoobscura*	0.79	0.35	0.31	0.41	0.14
Epstein barrvirus	0.42	0.26	0.24	0.06	0.10
Xenopus tropicalis	0.68	0.43	0.45	0.07	0
Total	0.57	0.29	0.51	0.30	0.22

It can be seen from the table that the accuracy of BP-Adaboost algorithm is superior to other algorithms, although the accuracy of the rest 4 algorithms is higher in some specific species, the accuracy of the algorithm in this study is the highest in total.

## Discussions

In this paper, we proposed a new method to identify the pre-miRNAs. We use the Adabost algorithm to generate ten BP classifiers to finish the identification. It can provide a new thinking of solving the problem of miRNAs identification. We use several original algorithms to compare with our method, and found that our method can achieve better performance than them. Although the method can fully play the generalization of BP, and make the whole method hardly over-fit, it still has the problems such as: its performance is not ideal in solving multi-classification problem with imbalanced samples, its training time is longer than normal BP algorithm. In the respect of experiment, the method should be tested in many other data sets to verify the effectiveness of BP-Ababoost.

## Conclusions

The identification of miRNAs is significant for human to study its function and understand its network regulation mechanism, discovering more novel miRNAs can also promote the prediction of miRNA target genes and the development of new drugs. In this study, we proposed a method combined BP neural network with Adaboost algorithm, it can effectively overcome the defects of unstable output and overfitting phenomenon, our method obtained a strong classifier by integrating multiple week classifiers (BP neural network classifiers) and distributing the weights to them. The data set of traditional classification of real and pseudo pre-miRNA sequences combined the real and pseudo sequences of one species together, in this study, we combined the real and pseudo sequences of 7 different species together, which increased the diversity and difficulty of the classification. In the end, we obtained a high accuracy identification result of real and pseudo pre-miRNAs. Beyond that, in this study we also classified 7 different species from pre-miRNAs which is the part that few people are paying attention to. Due to that, the sample data is not enough, though the accuracy of our classifier is higher than other methods, but the overall classification result is still need to be proved. However, the method we proposed is still able to provide guidance for the miRNA identification.

## Abbreviations

ACC: Accuracy rate
BP-Adaboost: Back Propagation neural network fused with Adaboost algorithm
FN: False Negative
FP: False Positive
miRNA: microRNA
MSE: Mean Square Error
RF: Random Forest algorithm
SVM: Support Vector Machine algorithm
TN: True Negative
TP: True Positive
